# When neuroprotection fails early: prokineticin downregulation in idiopathic REM sleep behavior disorder

**DOI:** 10.1093/sleep/zsag024

**Published:** 2026-02-02

**Authors:** Gabriele Bellini, Domeniko Hoxhaj, Giovanni Palermo, Roberto Ceravolo, Michelangelo Maestri Tassoni

**Affiliations:** Neurology Unit, Department of Clinical and Experimental Medicine, University of Pisa, Pisa, Italy; Department of Neurology, The Marlene and Paolo Fresco Institute for Parkinson's and Movement Disorders, NYU Langone Health, New York City, NY, United States; Neurology Unit, Department of Clinical and Experimental Medicine, University of Pisa, Pisa, Italy; Neurology Unit, Department of Clinical and Experimental Medicine, University of Pisa, Pisa, Italy; Neurology Unit, Department of Clinical and Experimental Medicine, University of Pisa, Pisa, Italy; Center for Neurodegenerative Diseases, Department of Clinical and Experimental Medicine, University of Pisa, Pisa, Italy; Neurology Unit, Department of Clinical and Experimental Medicine, University of Pisa, Pisa, Italy

Idiopathic rapid eye movement sleep behavior disorder (iRBD) is widely recognized as one of the most specific prodromal manifestations of α-synucleinopathies [1]. Recent meta-analytic evidence has confirmed the exceptionally high risk of phenoconversion associated with iRBD. Among individuals with video-polysomnography-confirmed iRBD, approximately one-third convert to a defined α-synucleinopathy within the first 5 years following diagnosis [2]. This risk increases steeply over time, reaching about 82% after 10 years and exceeding 95% after more than a decade of follow-up [3]. Taken together, these data indicate that iRBD represents a common prodromal biological substrate from which distinct α-synucleinopathy phenotypes may later emerge. Because of its high and time-dependent probability of conversion, iRBD can be conceptualized as an early “sentinel node” of underlying α-synuclein pathology. This unique position distinguishes iRBD from other prodromal features and makes it a uniquely informative biological window for investigating molecular and cellular mechanisms that operate before widespread neurodegeneration. In the paper, Grillo and colleagues report a striking downregulation of the prokineticin system in olfactory neurons from individuals with iRBD, providing novel evidence that failure of endogenous neuroprotective pathways may characterize the prodromal phase of α-synucleinopathies [4].

Prokineticin-2 (PROK2) is a chemokine-like peptide involved in neuroprotection, mitochondrial homeostasis, and regulation of neuroinflammatory responses. Experimental models of Parkinson’s disease (PD) have consistently shown upregulation of PROK2 in dopaminergic neurons in response to neurotoxic stress. It engages its G-protein–coupled receptors PKR1 and PKR2 to activate ERK1/2 and PI3K/Akt pathways, promoting resistance to oxidative stress, preserving mitochondrial integrity, and limiting apoptotic cascades, including caspase-3 activation, in response to neurotoxic insults [5, 6]. Beyond acute survival signaling, PROK2 has been shown to enhance mitochondrial biogenesis through PGC-1α, thereby improving energetic resilience in vulnerable nigrostriatal neurons [4, 5, 7]. In parallel, PROK2 enhances glutamate clearance by upregulating the astrocytic transporter GLAST, thereby reducing excitotoxic stress, a key contributor to dopaminergic neuron vulnerability [8]. Moreover, PK2-driven polarization toward a protective A2 astrocytic phenotype promotes the release of neurotrophic factors, including GDNF, further supporting dopaminergic neuron survival and reinforcing a glia-mediated neuroprotective environment in experimental models of PD [8]. Through these convergent mechanisms, PROK2 functions as an endogenous, stress-responsive signaling hub that links neuronal injury to adaptive, multicellular protective responses.

In humans, increased PROK2 expression has been reported in the substantia nigra, serum, olfactory neurons, and colonic mucosa of patients with established PD (Table 1), supporting the concept that the prokineticin system acts as a compensatory defensive response once neurodegeneration is underway [7, 9, 10]. As summarized in Table 1, these findings have been replicated using heterogeneous methodological approaches, ranging from fluorescent Western blotting and immunohistochemistry in post-mortem brain samples, to ELISA-based quantification in serum, and combined gene expression and protein localization analyses in olfactory neurons and colonic mucosa. Notably, the convergence of these observations across central, peripheral, and enteric compartments suggests that PROK2 modulation reflects a systemic biological response rather than a region-specific epiphenomenon.

**Table 1:** Human evidence on prokineticin-2 dysregulation in Parkinson’s disease

Study (year)	Population	Tissue/matrix	PD stage	PK2 assessment method	PK2 change
Gordon et al. [5]	Post-mortem brain samples of PD (*n* = 10) vs healthy controls (*n* = 10)	Substantia nigra	Neuropathologically confirmed PD (post-mortem) (*n* = 10)	Fluorescent Western blot + densitometry; DAB IHC; PK2/TH co-labelling	↑ Increased in PD
Schirinzi et al. [10]	PD patients (*n* = 31) vs healthy controls (*n* = 14)	Serum	PD (*n* = 31)	ELISA(serum PK2 concentration)	↑ Increased in PD
Schirinzi et al. [9]	PD patients (*n* = 38) vs healthy controls (*n* = 31)	Olfactoryneurons	De novo PD (*n* = 26) and advanced PD (*n* = 12)	RT-qPCR (gene expression), immunofluorescence (proteinlocalization/intensity)	↑ Increased in PD; higher in de novo than in advanced PD
Bellini et al. [7]	PD patients (*n* = 11) vs healthy controls (*n* = 5)	Colonic mucosa	Early (<5 years, *n* = 5) and late (>5 years, *n* = 6) PD	Immunofluorescence (quantitative image analysis), Western blot (densitometry)	↑ Increased in early PD

The table summarizes post-mortem and in vivo human studies assessing PK2 expression in PD across central and peripheral tissues, including substantia nigra, serum, olfactory neurons obtained by nasal brushing, and colonic mucosal biopsies. For each study, the experimental population, tissue or biological matrix analyzed, disease stage, analytical method used to assess PK2, and direction of change relative to healthy controls are reported. Where available, subgroup analyses according to disease stage (e.g. de novo vs advanced PD, or early vs late disease duration) are specified. Direction of PK2 change refers to relative differences between PD patients and control subjects as reported in the original studies. PD, Parkinson’s disease; PK2, prokineticin-2; TH, tyrosine hydroxylase; IHC, immunohistochemistry; DAB, 3,3′-diaminobenzidine; ELISA, enzyme-linked immunosorbent assay; RT-qPCR, real-time quantitative polymerase chain reaction.

Moreover, where disease stage has been examined, PROK2 elevation appears most prominent in early or de novo PD, with attenuation or loss of increase in more advanced stages, further supporting the interpretation of PROK2 upregulation as an early, and potentially time-limited, adaptive response to neurodegenerative stress [4, 6–8].

Whether this pathway is already engaged during prodromal disease stages, however, has remained unknown.

Grillo et al. directly address this gap by examining olfactory neurons obtained via non-invasive nasal brushing in a carefully characterized cohort of video-polysomnography-confirmed iRBD subjects [4]. This approach is particularly compelling, as the olfactory system represents one of the earliest sites of α-synuclein pathology and is anatomically and biologically linked to central neurodegenerative processes [11–14]. Using complementary molecular and protein-based techniques, the authors demonstrate reduced PROK2 protein levels in olfactory neurons and serum from iRBD individuals, accompanied by decreased mRNA expression of both prokineticin receptors. Importantly, these changes occur in the absence of a robust accumulation of oligomeric α-synuclein, which showed only a non-significant trend toward increase [4].

This dissociation suggests that prokineticin downregulation is not merely a downstream consequence of overt α-synuclein aggregation, but may instead reflect an early impairment of intrinsic neuroprotective capacity. Such an interpretation aligns with emerging evidence that prodromal PD is characterized not only by neurodegenerative processes, but also by early failure of immune and stress-response mechanisms. Reduced adaptive immune activity, altered lymphocyte profiles, and diminished humoral responses to α-synuclein have all been reported years before PD diagnosis, supporting the idea that loss of resilience may precede and facilitate neurodegeneration [15–17].

Within this framework, the findings by Grillo et al. support a biphasic model of prokineticin involvement across the α-synucleinopathy spectrum [4]. In the prodromal phase, exemplified by iRBD, the prokineticin system appears downregulated, potentially lowering the threshold for neuronal vulnerability. As pathology progresses and α-synuclein burden and inflammatory stress increase, prokineticin signaling may subsequently become upregulated, as observed in early and established PD, representing a delayed and ultimately insufficient compensatory response [7, 9, 10]. Longitudinal studies, evaluating neurodegenerative trajectories of iRBD patients, would help us to understand this hypothesis regarding prokineticin system changes over time and in different clinical subsets. Moreover, this biphasic model of protective systems, and not just a linear progression from iRBD to PD as revealed in most of the clinical or molecular biomarkers, suggests a more complex dynamics in the progression of the neurodegenerative process than the one generally supposed.

Beyond its mechanistic implications, the work by Grillo et al. also carries translational relevance. The detection of reduced PROK2 levels in serum, albeit in a limited subsample, raises the possibility that components of the prokineticin pathway could serve as accessible peripheral biomarkers of prodromal neurodegeneration [4]. As clinical trials increasingly focus on treating PD and iRBD at their earliest stages, identifying biomarkers that reflect early breakdown of protective mechanisms may be just as important as detecting abnormal protein accumulation itself [18–21].

Finally, the authors address possible confounding effects related to sleep itself. Although PROK2 is involved in circadian signaling in the suprachiasmatic nucleus [22, 23], it is well known that circadian and homeostatic sleep regulation are preserved in iRBD, and PROK2 is not linked to REM atonia mechanisms. These findings support a disease-related interpretation, suggesting that PROK2 dysregulation reflects prodromal neurodegenerative processes rather than sleep disturbance itself.

In sum, Grillo et al. provide compelling evidence that iRBD is marked by early downregulation of a key neuroprotective pathway, shifting the focus from toxic drivers alone to the premature failure of endogenous defense mechanisms. This work not only deepens our understanding of prodromal α-synucleinopathies but also opens new avenues for biomarker development and therapeutic strategies aimed at restoring resilience before irreversible neurodegeneration takes hold [4].
